# Infodemic vs. Pandemic Factors Associated to Public Anxiety in the Early Stage of the COVID-19 Outbreak: A Cross-Sectional Study in China

**DOI:** 10.3389/fpubh.2021.723648

**Published:** 2021-08-30

**Authors:** Jian Xu, Cong Liu

**Affiliations:** ^1^School of Media and Communication, Shanghai Jiao Tong University, Shanghai, China; ^2^China Institute for Urban Governance, Shanghai Jiao Tong University, Shanghai, China; ^3^Institute of Cultural Innovation and Youth Development, Shanghai Jiao Tong University, Shanghai, China

**Keywords:** infodemic, COVID-19, anxiety, information overload, vicarious trauma, commercial media, urban governance

## Abstract

**Introduction:** Every outbreak of an epidemic or pandemic disease is accompanied by the tsunami of information, which is also known as the infodemic. Infodemic makes it hard for people to find trustworthy sources and reliable guidance when they need it, and causes social panic about health, widens the gaps between races and regions, and even brings the social chaos all over the world. While most researchers and related parties made efforts to control the inaccurate information spreading online during the COVID-19 pandemic, the infodemic influence caused by the overload of accurate information were almost or completely ignored, and this will hinder the control of infodemic in future public health crises. This study aims to explore the infodemic vs. pandemic influence on people's psychological anxiety across different media sources in the early stage of the COVID-19 outbreak in China.

**Methods:** A cross-sectional study using online survey method was conducted by a data-collection service provider in April 2020. A total of 1,117 valid samples were finally collected from 5,203 randomly invited members *via* webpages and WeChat. The sample distribution covered the 30 provincial administrative divisions of mainland China.

**Results:** Hierarchical regression analysis for the potential pandemic sources and infodemic sources of psychological anxiety showed that the infodemic factors of attention to the coronavirus information (β = 0.154, *p* < 0.001) and commercial media exposure (β = 0.147, *p* < 0.001) is positively related to the level of anxiety. Statistics indicated that influence of the infodemic factors is over and above that of the pandemic factors (Δ*R*^2^ = 0.054, *F* = 14.199, and *p* < 0.001). Mediation analysis showed that information overload (*B* = 0.155, Boot SE = 0.022, and 95% Boot CI [0.112, 0.198]) mediates the link between attention to coronavirus information and anxiety; both information overload (*B* = 0.035, Boot SE = 0.014, and 95% Boot CI [0.009, 0.062]) and media vicarious traumatization (B = 0.106, Boot SE = 0.017, and 95% Boot CI [0.072, 0.140]) mediate the link between commercial media exposure and anxiety.

**Conclusion:** This study suggested that the influence of infodemic with mixed accurate and inaccurate information on public anxiety does exist, which could possibly go beyond that of the pandemic. Information overload and vicarious traumatization explain how infodemic may be associated to public anxiety. Finally, commercial media could be a major source of infodemic in the Chinese media context. Implications for the related parties were discussed.

## Introduction

### Background

At the beginning of 2020, the whole world fell into an emergent public health crisis brought by the COVID-19 pandemic. Accompanied with the outbreak of the COVID-19 was the tsunami of the disease-related information, which is known as the infodemic (1). In this crisis, media plays an important role in people's information acquisition. All media outlets were unprecedentedly active, reaping countless high searching, reading, and forwarding volumes. However, some media outlets have also been criticized for providing false information, inflammatory views, and even unethical content. Although the pandemic has been under well-control in some countries, a series of social, psychological, and ethical issues brought about by the infodemic still worth to be reconsidered.

Infodemic is a phenomenon described as an over-abundance of information—some accurate and some not—that makes it hard for people to find trustworthy sources and reliable guidance when they need it (2). The World Health Organization declared that besides the pandemic threat originated by the COVID-19 virus, an infodemic has been generated by a large amount of information available on the matter, as well as by the difficulty to sort the veracious information from the false (2). Although the outbreak of SARS in 2003, H1N1 in 2009, and MERS in 2012 were all accompanied by rumors and false information, creating different degrees of social panic, the COVID-19 pandemic has developed unprecedented trend of infodemic with the emerging information technology, which was defined as “the first true social-media infodemic” (3). Social media is considered a powerful tool for sharing health information related to pandemic risks (4, 5). After the COVID-19 outbreak, 70–80% of the Chinese users reported an increase in the use of WeChat (6, 7).

Infodemic, including dissemination of conflicting or unclear messages, misinformation, rumors, and conspiracy theories, can profoundly cause public anxiety and social panic, affect public health communication, diminish preventive measures, impede effective crisis management, widen the gaps between races and regions, and even bring the social chaos all over the world (8–12). The heightened distress by the infodemic can also cause individuals' irrational behaviors in the crisis, such as health information avoidance, spread of misinformation, overuse of the healthcare services, panic purchases, incompliance with preventive measures (such as physical distancing, mask wearing, and vaccination) (13–17). The World Health Organization, the United Nations, the United States Centers for Disease Control and Prevention, and many other organizations had all engaged in fighting against the infodemic by debunking of false information, stopping the spread of rumors, providing the population with reliable data and updated news about COVID-19 (18–20). Technology industries including Google, Amazon, Facebook, YouTube, Microsoft, and Twitter also implemented restrictions on publishing pandemic-related content and removed medically disproved claims (21–24).

Interesting enough, existing studies seem suggest that online users have an adequate e-health literacy and can effectively filter the false information in emergent public health crises. Studies showed that users can critically evaluate the source of the health information received and are capable to discriminate between reliable and unreliable content, and they place higher trust in the medical professionals and scientists than the mass media and social media, and they also rated the authorities' social media channels as more trustworthy than the user-generated content (25, 26). Big data analysis also showed that information from questionable sources or false information posted on social media only represents a small fraction compared to the reliable or science-based ones (27). Researchers claimed that there is a higher potential of true information to capture more user engagement (28). True information was also found to circulate more, reach a higher level of diffusion, spread more quickly, and have a longer lifetime than false information (29, 30). These suggest that false information dissemination may not be the only cause of infodemic and public anxiety.

### Infodemic vs. Pandemic Factors of Public Anxiety

Previous studies have shown that people generally have varying degrees of anxiety in the context of public health emergencies. Uncertainty situations make people more vulnerable to mental and psychological distress. In the early stage of the COVID-19 outbreak, especially in China, people were exposed to unknown threats, and highly uncertain about the infectivity, susceptibility, and treatment methods of the COVID-19. A study showed that more than half of the Chinese participants suffered from psychological distress, anxiety, depression, and stress at moderate to severe levels (31). Perceived risk of infection is one of the most direct factors that cause individual anxiety and fear. Studies have revealed that perceived risk of COVID-19 significantly associated with anxiety-related feelings such as sleeping disorder, stress, worry, and disruption of daily life (32–34).

Besides the pandemic factors, public anxiety levels are also largely influenced by the infodemic factors, especially in the highly developed information technology era. Researchers found that the excessive social media use leads to increased levels of stress, anxiety, and vicarious trauma (35). An online survey indicated a positive link between information exposure during the COVID-19 pandemic and the occurrence of anxiety and insomnia symptoms, and the strength of the association increases with the duration of the media exposure (12, 36). Major information sources of the COVID-19 pandemic not only involve social media, but also traditional media (12). Experts and scholars criticized that social media as well as traditional mass media were disseminating inaccurate information during the COVID-19 pandemic, and even most extreme pictures found elsewhere sending the wrong message were being used in many mainstream newspapers and TV reports (9).

### Information Overload

Information overload represents a state in which an individual's efficiency in using information in their work is hampered by the amount of relevant, and potentially useful, information available to them. The feeling of overload is usually associated with a loss of control over the situation, and sometimes with feelings of being overwhelmed (37). Information is a double-edged sword in the COVID-19 pandemic. On the one hand, effective communication of facts helps people to obtain adequate risk perceptions and make adaptive health behavior, while on the other hand, overloaded information can also impose strains on crisis management (26, 38, 39). Studies showed that as people are intensively exposed to negative information about a crisis, their levels of anxiety and other unpleasant emotions could be triggered and elevated for an extended period (40–43), especially when their personal experience with the disease is limited (25, 44, 45).

### Media Vicarious Traumatization

Vicarious trauma describes the trauma experiences people have after being exposed to others' trauma stories and having witnessed the pain, fear, and terror that traumatized survivors have endured (46, 47). Media could be another source of vicarious trauma (48), when audiences indirectly experience the traumatic events *via* the vividly presented videos, pictures, and texts exposed by the media. Studies showed that obtaining more informational support *via* social media increased users' vicarious trauma levels (35). When the information and media content are perceived as threatening, aversive emotions can be elicited (49–52), and when information is contradictory or uncertain, the distress may be even more elevated (53, 54). Extensive research indicated that consuming media coverage to the natural or humanmade disasters typically associates with increased incidences of post-traumatic stress disorder (PTSD), anxiety, and depression (55–58).

### Goal of This Study

Most researchers and related organizations engaged in dealing with the control of false information dissemination online (such as misinformation, fake news, rumors, conspiracy theories) as well as their negative influence on public mental health and health related behaviors during the COVID-19 pandemic. However, a key question remained was that “is the false information spreading the only cause of public anxiety?” A critical part was almost or completely ignored in the existing studies and countermeasures, which is, the infodemic caused by the over-abundance of the mixed inaccurate and accurate information disseminated by the social media, mass media, and even government official media.

This study will focus on the influence of infodemic across different media sources on people's anxiety in the early stage of the COVID-19 outbreak in China. It aims to answer the following three research questions:

(a) will the infodemic factors vs. pandemic factors significantly associated with people's anxiety?
(b) what are the underlying mechanisms of the impact of infodemic on people's anxiety; that is, how information overload and media vicarious traumatization mediate the impact?
(c) what are the roles played by the three main information sources in the Chinese media context (i.e., government official media, commercial media, and social media) in the infodemic?

## Methods

### Recruitment

The data were collected online by a sample service provider (i.e., Changsha Ranxing IT Ltd.), who owns one of the biggest online sample with more than 2.6 million members all over China. The survey was conducted by randomly inviting 5,203 members from the 30 provinces of mainland China *via* webpages and WeChat in April, 2020. A total of 1,342 members responded to the invitation. Among them, 225 invalid responses were systematically or manually eliminated by the sample service provider, and the final valid responses received were 1,117 with a response rate of 21.5%. Cities mostly influenced by the COVID-19 in the early stage of the outbreak were all covered, and the regional distribution of the samples were as follows: Wuhan (9%) and other cities (14%) of Hubei Province; Guagnzhou (6%) and Shenzhen (6%) of Guangdong Province; Wenzhou (7%) of Zhejiang Province; Beijing (7%); Shanghai (6%); Chongqing (7%); and other cities of the other 24 provinces (38%). Participation of the survey was anonymous and voluntary.

### Ethical Consideration

The protocol of this study was approved by the Institutional Review Board of Shanghai Jiao Tong University (No: H2020038I). The data were treated with confidentiality and the results did not identify the participants personally.

### Participants

Less than half of the participants are male (45.9%) and 54.1% are female. A majority age between 18 and 40 years old (85.5%). Almost all of them are in good health condition (98%). During the pandemic, 23.0% of the participants stayed in Hubei Province, and 77% stayed in other provinces; most of the participants stayed with family members or friends (97.7%), and only 2.3% stayed alone. Detailed participant characteristics are shown in Table 1.

**Table 1:** Characteristics of the participants.

**Demographics**	**Percentage**	***n***
**Gender**		
Male	45.9%	513
Female	54.1%	604
**Age**		
<18	4.1%	46
18–25	30.6%	342
26–30	22.6%	252
31–35	23.5%	263
36–40	9%	101
41–50	7.6%	85
>50	2.5%	28
**Health condition (Mean = 3.92, SD = 0.72)**		
Very poor	0.1%	1
Relatively poor	2.0%	22
Average	24.0%	268
Relatively good	53.6%	599
Very good	20.3%	227
**Place of residence**		
Hubei province	23.0%	257
Other provinces	77.0%	860
**Accommodation**		
Stay alone	2.3%	26
Stay with family/friends	97.7%	1,091

*N = 1,117*.

*SD, standard deviation*.

### Measurements

#### Psychological Anxiety

The psychological anxiety questionnaire was adapted from Zung's Self-Rating Anxiety Scale (SAS) (59). Participants were asked to rate their level of anxiety in the early stage of the COVID-19 outbreak on a 5-point Likert scale (1 = completely disagree, 5 = completely agree) with three items, including “I feel nervous and anxious due to the coronavirus pandemic,” “I have sleeping problems during the coronavirus pandemic,” and “I feel panicky and cannot sit still easily during the coronavirus pandemic.” Higher scores indicate higher levels of psychological anxiety. Cronbach's alpha of this questionnaire is 0.83. Correlations between items 1 through 3 with the total score are 0.85 (*p* < 0.01), 0.88 (*p* < 0.01), and 0.87 (*p* < 0.01), respectively.

#### Pandemic Factors

Participants were asked to rate their perceptions to the COVID-19 pandemic in the early stage of the outbreak. Four indicators were adopted from the widely used measurements for the Health Belief Model variables (60, 61): (a) perceived risk of oneself getting infected by coronavirus (from 0 to 100%), (b) perceived risk of people around getting inflected by coronavirus (from 0 to 100%), (c) worry about oneself getting infected by coronavirus (1 = not at all, 5 = very much), and (d) worry about people around getting infected by coronavirus (1 = not at all, 5 = very much).

#### Infodemic Factors

Participants were asked to rate their information consumption in the early stage of the COVID-19 outbreak, which includes five indicators: attention to coronavirus information and attention to information irrelevant to the coronavirus on 5-point Likert scales (1 = hardly ever, 2 = less than an hour, 3 = 1–3 h, 4 = 3–5 h, 5 = more than 5 h); exposure to different media sources including the government official media (e.g., CCTV, People's Daily, Hubei Daily), commercial media (e.g., The Paper, Sanlian Life Week, Caixin), and social media (e.g., WeChat, Weibo, TikTok) on 5-point Likert scales (1 = never, 5 = often).

#### Information Overload

Information overload was measured by the questions adapted from Zhang and colleagues' Information Overload Questionnaire on a 5-point Likert scale (1 = completely disagree, 5 = completely agree) including five items, for example, “I find that only a small part of the coronavirus information is relevant to my needs,” “I find that I am overwhelmed by the amount of coronavirus information I have to process on a daily basis,” and “There is too much information so I find it a burden to handle” (62). Cronbach's alpha of this questionnaire is 0.76.

#### Media Vicarious Traumatization

Media vicarious traumatization was measured by the questions adapted from the Vrklevski's Vicarious Traumatization Scale (VTS) on a 5-point Likert scale (1 = completely disagree, 5 = completely agree) with seven items, including “I was exposed to distressing news and experiences about coronavirus *via* media,” “It is hard to stay positive and optimistic given some of the coronavirus information I get from the media,” and “I find myself thinking about distressing coronavirus news on media” (63). Cronbach's alpha of this questionnaire is 0.78.

### Statistical Analysis

All statistical analysis were performed using SPSS statistics (v25, IBM, USA). Descriptive analysis concerning the minimums, maximums, means, and standard deviations of the main variables were reported. To examine the influences of the pandemic and infodemic factors on anxiety, a hierarchical regression analysis was conducted including the control variables (i.e., gender, age, health condition, accommodation, and place of residence), the pandemic factors (i.e., risk of oneself, risk of people around, worry about oneself, worry about people around), and the infodemic factors (i.e., attention to coronavirus information, attention to coronavirus-irrelevant information, government official media exposure, commercial media exposure, and social media exposure) in three blocks, respectively. Improvements in model fit was indicated by the *R*^2^ change in each block. To analyze the underlying mechanisms of the influence of the infodemic factors, mediational analyses using Process Macro model 4 (64) were conducted. Direct and indirect effects were reported with their 95% confidence intervals.

## Results

### Descriptives

Descriptives of the statistics are shown in Table 2. Psychological anxiety of the participants in the early stage of the pandemic is relatively low with the mean score of 2.83 out of 5. Among the pandemic factors, the average perceived risk of oneself getting infected is 41.67%, and the average perceived risk of people around getting infected is 44.25%. The mean score of worry about oneself getting infected is 3.49, and the mean score of worry about people around getting infected is 3.38. Among the infodemic factors, the mean score of attention to coronavirus information is 3.02, and the mean score of attention to coronavirus-irrelevant information is 2.87. Media sources exposed to the participants from the most frequent to the least frequent are social media (mean score is 4.24), government official media (mean score is 4.01), and commercial media (mean score is 2.73). The mean score of information overload is 2.97. The mean score of media vicarious traumatization is 3.27.

**Table 2:** Descriptives of the main variables.

	**Minimum**	**Maximum**	**Mean**	**SD**
**Psychological anxiety**	1	5	2.83	0.98
**Pandemic factors**				
Risk_oneself	0	100	41.67	17.71
Risk_people around	0	100	44.25	20.61
Worry_oneself	1	5	3.49	0.71
Worry_people around	1	5	3.38	0.84
**Infodemic factors**				
Attention_coronavirus information	1	5	3.02	0.83
Attention_coronavirus-irrelevant information	1	5	2.87	1.01
Government official media	1	5	4.01	1.10
Commercial media	1	5	2.73	1.15
Social media	1	5	4.24	0.93
**Information overload**	1	5	2.94	0.79
**Media vicarious traumatization**	1.43	5	3.27	0.66

*N = 1,117*.

*SD, standard deviation*.

### Regression Analysis for the Pandemic and Infodemic Factors of Anxiety

A hierarchical regression was conducted to analyze the pandemic and infodemic factors on psychological anxiety, with the control variables entered to the first block, the pandemic factors entered to the second block, and the infodemic factors entered to the third block. Results (see Table 3) showed that among the control variables, age (β = 0.095, *p* < 0.01), health condition (β = −0.148, *p* < 0.001), and accommodation (β = 0.075, *p* < 0.05) are significantly correlated to anxiety. In specific, participants who are older, in poorer health condition, or staying alone are more anxious than their counterparts. Gender and place of residence are not correlated to anxiety. Variance explained by the control variables (Δ*R*^2^) is 0.043 (*F* = 9.870, *p* < 0.001). Among the pandemic factors, perceived risk of oneself getting infected (β = 0.094, *p* < 0.01) and worry about oneself getting infected (β = 0.180, *p* < 0.001) are positively correlated to anxiety after controlling for the effects of the control variables, while perceived risk of people around and worry about people around getting infected are not significantly correlated to anxiety. Variance uniquely explained by the pandemic factors (Δ*R*^2^) is 0.062 (*F* = 19.073, *p* < 0.001). Among the infodemic factors, attention to the coronavirus information (β =0.154, *p* < 0.001) and commercial media exposure (β = 0.147, *p* < 0.001) are positively related to anxiety after controlling for the effects of the control variables and the pandemic factors, while attention to coronavirus-irrelevant information, government official media exposure, and social media exposure are not significantly related to anxiety.

**Table 3:** Herarchical regression for the pandemic and infodemic factors of anxiety.

	***B***	**SE**	**β**	***t***	***p***
**Control variables**					
Gender	0.079	0.058	0.040	1.366	0.172
Age	0.065	0.020	0.095^**^	3.180	0.002
Health condition	−0.201	0.040	−0.148^***^	−5.012	0.000
Accommodation	0.485	0.191	0.075^*^	2.541	0.011
Place of residence	0.058	0.069	0.025	0.844	0.399
Δ*R*^2^ = 0.043 (*F* = 9.870, *p* < 0.001)					
**Pandemic factors**					
Risk_oneself	0.003	0.001	0.094^**^	2.622	0.009
Risk_people around	0.000	0.001	−0.011	−0.295	0.768
Worry_oneself	0.125	0.025	0.180^***^	5.022	0.000
Worry_people around	0.023	0.022	0.039	1.048	0.295
Δ*R*^2^ = 0.062 (*F* = 19.073, *p* < 0.001)					
**Infodemic factors**					
Attention_coronavirus information	0.182	0.035	0.154^***^	5.174	0.000
Attention_coronavirus-irrelevant information	−0.023	0.028	−0.024	−0.849	0.396
Government official media	−0.016	0.027	−0.018	−0.598	0.550
Commercial media	0.126	0.026	0.147^***^	4.874	0.000
Social media	0.044	0.030	0.042	1.479	0.140
Δ*R*^2^ = 0.054 (*F* = 14.199, *p* < 0.001)					

*N = 1,117*.

* *p < 0.05*,

** *p < 0.01*,

*** *p < 0.001*.

*B, unstandardized regression coefficient; SE, standard error; β, standardized regression coefficient; t, value of the t-test statistic; p, probability; ΔR^2^, multiple correlation squared changed; F, Fisher's F ratio*.

The hierarchical regression analysis showed that variance uniquely explained by the infodemic factors (Δ*R*^2^) is 0.054 (*F* = 14.199, *p* < 0.001). It also indicated a unique contribution of the infodemic factors on anxiety over and above that of the pandemic factors. In other words, statistics supports that the influence of the infodemic factors are beyond that of the pandemic factors in increasing psychological anxiety.

### Mediational Analysis of Information Overload and Media Vicarious Traumatization

Since the above analysis indicates that attention to coronavirus information and commercial media exposure are the two key infodemic factors, the underlying mechanisms of these two factors was further explored with mediational analyses.

**Figure 1:**
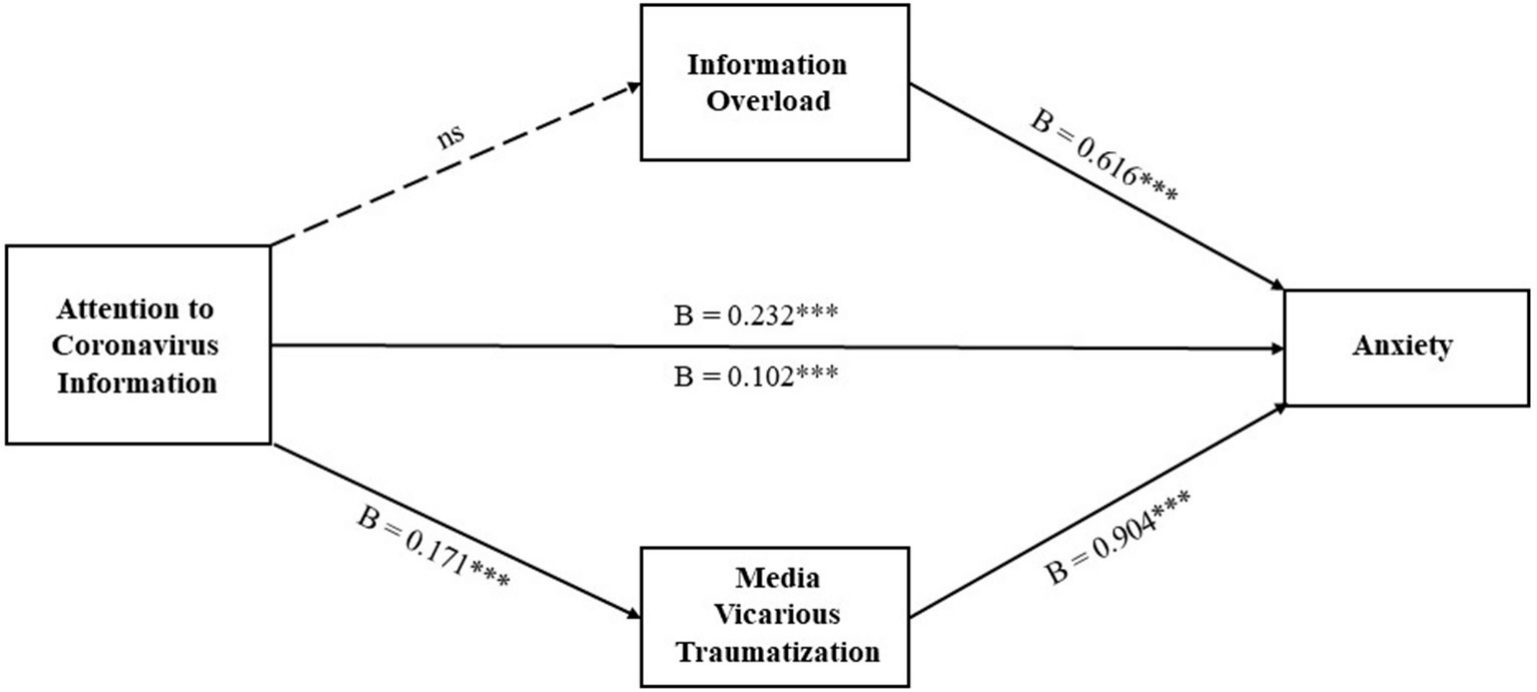
Mediational analysis for the effect of attention to coronavirus information on anxiety. B, unstandardized regression coefficient; ***p < 0.001; ns, nonsignificant.

A mediational analysis was firstly conducted for the effect of attention to coronavirus information on anxiety (see Figure 1). Results showed that when information overload is treated as the mediator, the mediation effect is not significant (B = 0.025, Boot SE = 0.018, and 95% Boot CI [−0.011, 0.059]), and there is only a direct effect of attention of coronavirus information on anxiety (B = 0.232, SE = 0.030, and 95% CI [0.174, 0.290]). When media vicarious traumatization is treated as the mediator, the mediation effect is significant (B = 0.155, Boot SE = 0.022, and 95% Boot CI [0.112, 0.198]), and the direct effect of attention of coronavirus information on anxiety is also significant (B = 0.102, SE = 0.028, and 95% CI [0.047, 0.157]). That is, the effect of attention to coronavirus information on anxiety is mediated by media vicarious traumatization.

**Figure 2:**
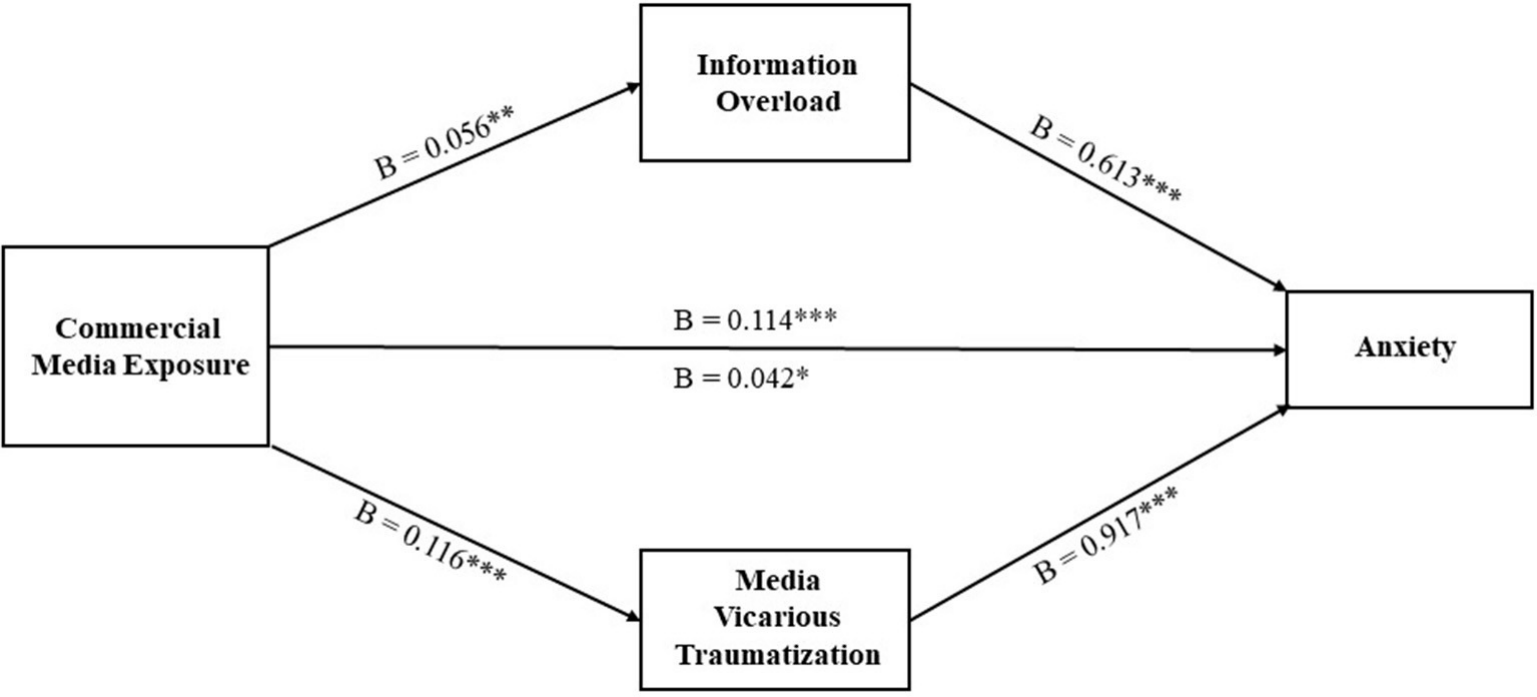
Mediational analysis for the effect of commercial media exposure on anxiety. B, unstandardized regression coefficient; ***p < 0.001, **p < 0.01, and *p < 0.05; ns, nonsignificant.

A mediational analysis was secondly conducted for the effect of commercial media exposure on anxiety (see Figure 2). Results showed that when information overload is treated as the mediator, the mediation effect is significant (B = 0.035, Boot SE = 0.014, and 95% Boot CI [0.009, 0.062]), and the direct effect of commercial media exposure on anxiety is also significant (B = 0.114, SE = 0.022, and 95% CI [0.071, 0.157]). When media vicarious traumatization is treated as the mediator, the mediation effect is significant (B = 0.106, Boot SE = 0.017, and 95% Boot CI [0.072, 0.140]), and the direct effect of commercial media exposure on anxiety is also significant (B = 0.042, SE = 0.020, and 95% CI [0.003, 0.082]). That is, the effect of commercial media exposure on anxiety is mediated by both information overload and media vicarious traumatization.

## Discussion

This study focuses on the infodemic vs. pandemic influence on people's anxiety across different media sources in the early stage of the COVID-19 outbreak among the Chinese participants. In specific, it (a) explored the influence of infodemic vs. pandemic on people's anxiety, (b) explored the mediation effect of information overload and media vicarious traumatization, and (c) compared the differences in the roles of government official media, commercial media, and social media. Findings showed that pandemic factors of perceived risk of oneself getting infected and worry about oneself getting infected are positively related to the level of anxiety; infodemic factors of attention to coronavirus information and commercial media exposure are positively related to the level of anxiety; government official media exposure, social media exposure, and attention to coronavirus-irrelevant information were found to be the insignificant infodemic factors. More importantly, statistics also indicated that influence of the infodemic factors is beyond that of the pandemic factors. Mediation analysis testing the underlying mechanisms of the infodemic influence showed that vicarious traumatization mediates the effect of attention to coronavirus information on anxiety; both information overload and media vicarious traumatization mediate the effect of commercial media exposure on anxiety.

The findings first suggest that the infodemic influence on people's anxiety with mixed accurate and inaccurate information does exist, which could possibly be more profound than that of the pandemic itself. During an emergent public health crisis, people are more inclined to acquire information in order to alleviate the sense of uncertainty (65), however, findings suggest that paying too much attention to the crisis information and being intensively exposed to certain types of media content about the crisis may exacerbate the anxious and stressful feelings. Findings of this study also indicated that even distractions from the coronavirus-irrelevant information, including entertainment, games, and daily news, do not effectively alleviate the anxiety. Thus, this further supports experts' opinions that infodemic is not only caused by the spreading of false information or rumors, accurate information routinely spread by different media outlets could also become potential sources of infodemic. On the one hand, it is important to increase the speed and width of spreading of information and scientific evidence from trustworthy sources, such as the public health officials, medical professionals, scientists, verified social media accounts, official reports, etc. The most crucial and official information should be communicated by these credible groups, in order to effectively lower the emotional taxing of the crisis (25). On the other hand, active citizenship against the spread of false information should also be advocated, knowing that users have the potential to be trained to debunk false information through scientific literacy cultivation (28).

Information overload and media vicarious traumatization were found to be the important underlying mechanisms explaining why and how infodemic may be associated with anxiety. When individuals are intensively exposed to the crisis information, it is inevitable to vicariously experience the traumatic contents, which will in turn, increase their level of anxiety. This problem is more salient in the case of commercial media exposure compared to the government official media exposure and social media exposure. Commercial media may not only trigger the distressed perception by the overloaded amounts of reports, but also bring about vicarious traumatization. During the pandemic in China, commercial media circulated vast amount of coronavirus information intensively, and such information were further pushed to their users continuously with the utilization of artificial intelligence based on algorithms and historical data. Thus, consumers of commercial media may passively receive overloaded coronavirus information that probably carries traumatic contents. While researchers have proposed the empathic style of communication and personal experience sharing as the infodemic countermeasure (66), our study suggested that such style could be inappropriate considering the vicarious traumatization effect of the media coverage. On the contrary, media should convey information to the public without sensationalizing the situation or providing disturbing images and videos so as to prevent bringing emotional trauma to the public.

Comparisons across information sources showed that commercial media could be a major source of infodemic in the Chinese media context. Commercial media coverages could directly and indirectly cause public anxiety by overloaded information output and vicarious traumatization. Such impact exists among the commercial media more obviously than other types of media, and this could be explained by the market-oriented nature and the report genres of the commercial media. Commercial media in China intend to focus on those vivid cases from the microscopic perspective, and their story-telling feature could more easily trigger the traumatic feelings of the audience. In the early stage of the COVID-19 outbreak, the commercial media were inclined to cover the contents such as the situation of the first-line treatment, the patients' and families' stressful experience, and how the Wuhan citizens were expelled in other cities or countries, meanwhile, they tended to focus on the detailed and negative incisions from the patients, family members, and medical staff perspectives. Some typical examples include the article “Mother died in Wuhan isolation ward” released by Phoenix News on 28 January, 2020, the article “Wuhan Community under the pressure of epidemic: After the elderly died of high fever at home” released by Caixin on 29 January, 2020, and the article “When the hotel reception heard that I was from Wuhan, they immediately called the police” released by ThePaper on 28, January, 2020, and these articles went viral in only a few hours. In contrast, the government official media coverages in China are more neutral, macroscopic, and science-based, which mostly covered the authentic data, progress of the pandemic, and the government responses. Social media were usually found to be a major source of false information and rumors during crises in many studies, however, it is important to note that social media not only spread the crisis information, but also play a role in the health information support as well as social and emotional support from family members, friends, and significant others (67, 68). This could be the reason why social media was not found to be a source of infodemic in the current study. Thus, commercial media together with other media outlets should actively mitigate infodemic and public anxiety during public health crises by avoiding overloaded crisis information reports and detailed trauma-related content. Governments could also direct public health policies to address the impact of media portals in their routine spreading of information in times of pandemics rather than merely dealing with false information (66).

## Conclusion

This study gives insights for the in-depth understanding of the infodemic impact by analyzing the essential attributes of the infodemic from aspects of definition, information sources, communication mechanisms, and social psychological impact. The research findings provide valuable implications and suggestions for infodemic governance from the perspective of media practitioners, policy makers, and media consumers.

## Data Availability Statement

The raw data supporting the conclusions of this article will be made available by the authors, without undue reservation.

## Ethics Statement

The studies involving human participants were reviewed and approved by Shanghai Jiao Tong University. The patients/participants provided their written informed consent to participate in this study.

## Author Contributions

All authors listed have made a substantial, direct and intellectual contribution to the work, and approved it for publication.

## Conflict of Interest

The authors declare that the research was conducted in the absence of any commercial or financial relationships that could be construed as a potential conflict of interest.

## Publisher's Note

All claims expressed in this article are solely those of the authors and do not necessarily represent those of their affiliated organizations, or those of the publisher, the editors and the reviewers. Any product that may be evaluated in this article, or claim that may be made by its manufacturer, is not guaranteed or endorsed by the publisher.
